# Risk stratifying patients with non-varicosic upper gastrointestinal hemorrhage using the Glasgow-Blatchford score: A case series of 91 patients

**DOI:** 10.1016/j.amsu.2022.103778

**Published:** 2022-05-13

**Authors:** Houcine Maghrebi, Hazem Beji, Anis Haddad, Amine Sebai, Samia Safraoui, Maroua Hafi, Asma Laabidi, Mohamed Jouini, Montasser Jamel Kacem

**Affiliations:** a General Surgery Department, La Rabta Hospital Tunis, Tunisia; b Gastroenterology Department, La Rabta Hospital Tunis, Tunisia; c Pharmacology Department, La Rabta Hospital Tunis, Tunisia; d University of Tunis El Manar – Faculty of Medicine of Tunis, Tunisia

**Keywords:** Glasgow-Blatchford score, Upper gastrointestinal bleeding, Non-varicosic upper gastrointestinal hemorrhage, Prognosis

## Abstract

**Introduction:**

Non-variceal upper gastrointestinal hemorrhage (NVUGIH) often leads to systematic hospitalization and emergency endoscopy. However, in most cases, it does not constitute an immediate life threat. This study aimed to evaluate the Glasgow-Blatchford Score (GBS) in predicting the need for transfusions, and/or endoscopic or surgical treatments.

**Materials and methods:**

We conducted a retrospective monocentric study including 91 patients admitted in the general surgery department of the Hospital La Rabta Tunis for a NVUGIH.

Univariate analysis was performed with the Student *t*-test for continuous variables and with the Chi-square test for categorical variables. For a cut-off point of 9, we calculated the sensibility and the sensitivity of the GBS to predict the need for transfusions and/or hemostatic procedure.

**Results:**

During the study period, 91 patients were admitted for NVUGIH. Sixty-one patients (67%) were transfused. Seven patients (7.7%) underwent emergency surgery and two patients had endoscopic hemostasis.

The predictive factors for the use of transfusion and/or hemostasic treatments were: Age >50 years, ASA score, HR ≥ 90 bpm, pallor, Hb ≤ 9,5 g/dl, Urea ≥9,7 mmol/L.

For a cut-off of 9 points of the GBS, sensitivity was 85.71% and specificity 92.86%. The positive predictive value was 96%. The negative predictive value was 74%.

**Conclusion:**

The main interest of the GBS lies in dispatching the patients between intensive care units for therapeutic intervention (if GBS> = 9) and ordinary hospitalization for surveillance (if GBS <9). It then makes it possible to rationalize the management of patients with digestive hemorrhage to identify those requiring hospital treatments (transfusion, endoscopic treatment, or surgery).

## Highlights

• Non variceal upper gastrointestinal bleeding often leads to systematic emergency endoscopy.
• In most cases, bleeding does not constitute an immediate life threat.
• The Glasgow-Blatschford score can be reliable to predict the need for therapeutic intervention.
• For a score inferior to nine, patients can be admitted into an ordinary unit.

## 1 Introduction

Non-variceal upper gastrointestinal hemorrhage (NVUGIH) is bleeding of the digestive tract proximal to the ligament of Treitz. The incidence is estimated at 20–60/100.000 people [1]. It is particularly high among elderly patients with other systemic diseases [2]. NVUGIH is still characterized by important morbidity and mortality [3]. The prognosis may vary from mild to life-threatening [4]. It often leads to systematic hospitalization and emergency endoscopy, even though, in most cases, it does not constitute an immediate life threat. However, patients with severe bleeding may present severe complications and even death if they do not receive the appropriate treatment timely.

Therefore, a better stratification of complications' risk would optimize patients’ management and avoid abusive hospitalizations. Early diagnosis and precise categorization of patients with higher mortality risk and higher risk of re-bleeding may considerably improve the efficiency of medical treatment [5]. Many scoring systems have been proposed to stratify patients into high and low risk. The Glasgow-Blatchford Score (GBS) is one of the most used scores [6,7].To date, there is still no consensus on the therapeutic strategy for patients presenting NVUGIH [8].

This study aimed to evaluate the Glasgow-Blatchford Score (GBS) in predicting the need for transfusions, and/or endoscopic or surgical treatments.

## 2 Materials and methods

### 2.1 Patients

This was a retrospective case series study from January 01, 2018 to December 31, 2021 conducted at la Rabta Hospital in Tunis. It included all the patients admitted to our surgery department for NVUGIH.

The inclusion criteria were the following:

1. NVUGIH defined as hematemesis, melena, coffee grounds vomiting, and fresh blood vomiting.
2. Bleeding unrelated to varices confirmed by endoscopy.

Patients <18 years were excluded from the study.

### 2.2 Treatment

All patients had gastrointestinal endoscopy during the hospital stay. All patients were treated with proton pump inhibitors.

### 2.3 Data collection

Data collection was done at our general surgery department A of the hospital La Rabta in Tunis. For every patient, the following information were collected; gender, age, history of gastrointestinal bleeding, liver failure, cardiac failure, syncope, clinical symptoms, blood pressure, heart rate, laboratory findings, endoscopic diagnosis, transfusion, endoscopic treatment, surgical treatment, and death.

### 2.4 Analysis of GBS

The GBS was calculated for all the patients. The GBS is shown in Table 1.

**Table 1:** Glasgow Blatchford score.

Admission parameter	Score value
Urea (mg/dl)	
≥6.5 to < 8.0	2
≥8.0 to < 10.0	3
≥10.0 to < 25.0	4
≥25.0	6
**Haemoglobin (g/dl)**	
**Men**	
≥12.0 to < 13.0	1
≥10.0 to <12.0	3
<10.0	6
**Women**	
≥10.0 to <12.0	1
<10.0	6
**Systolic BP (mmHg)**	
100 to 109	1
90 to 99	2
<90	3
**Other parameters**	
**Pulse > 100 bpm**	1
**Melena at presentation**	1
**Syncope**	2
**Hepatic disease**	2
**Cardiac failure**	2

### 2.5 Statistical analysis

SPSS software (version 25.0, SPSS, Chicago, IL) was used for all statistical analyses. The demographic, epidemiologic, and clinical features were analyzed using a descriptive study (mean, percentage, and interquartile range).

Patients were divided into two groups:

• Group 1: patients who required transfusion and/or hemostatic procedure (endoscopic and/or surgical).
• Group 2: patients who required only medical treatment.

Univariate analysis was performed with the Student *t*-test for continuous variables and with the Chi-square test for categorical variables. For a cut-off point of 9, we calculated the specificity and the sensitivity of the GBS to predict the need for transfusions and/or hemostatic procedure.

The work has been reported in line with the PROCESS criteria [9].

## 3 Results

During the study period, 91 patients were admitted for NVUGIH. The mean age of patients was 59 years old (17–96). The sex ratio of men to women was 1.39:1.

Melena was the clinical feature in 47% of cases. Mean hemoglobin was 8.4 g/dL (2.6–15.9). Mean urea was 11.9 mmol/L (2- 42). All the patients had an endoscopic examination. The main lesions found are grouped in Table 2. A gastroduodenal ulcer was the most frequent finding in our patients.

**Table 2:** Main lesions found at the endoscopic examination.

Endoscopic lesions	Number of patients (N)	Percentage (%)
Peptic Ulcer	30	32.9
Esophagitis	7	7.7
Acute Duodenitis	11	12.1
Acute Gastritis	16	17.6
Malignant Lesion	4	4.4
Gastric Ulceration	9	9.9

The mean value of the Blachford score was 9 (0–19). Sixty-one patients (67%) were transfused. The mean number of pellets received by a patient was two (1–13). Seven patients (7.7%) underwent emergency surgery and two patients had endoscopic hemostasis. Hemostasis was obtained in 88 patients (96.7%). Five patients (5,49%) experienced bleeding recurrence.

The predictive factors for the use of transfusion and/or hemostasis gestures are summarised in Table 3.

**Table 3:** Predictive factors for the use of transfusion and/or hemostasis gesture.

Studied Variables	Group 1	Group 2	P
Epidemiological Variables			
**Age > 50 ans** Yes No	50 13	15 13	**0.002**
**Gender** Male Female	39 24	14 14	0.28
**ASA Score** ASA 1 ASA 2 or 3	13 50	16 12	**0.001**
**Elevated Blood Pressure** Yes No	23 40	9 19	0.68
**Known Peptic Ulcer** Yes No	5 58	1 27	0.66
**History of upper gastrointestinal haemorrage** Yes No	12 51	5 23	0.893
**Liver Disease** Yes No	4 59	0 28	0.173
**Heart Failure** Yes No	3 60	0 28	0.240
**Clinical Variables**			
**Heart Rate ≥ 90** Yes No	32 31	4 24	**0.001**
**Syncope** Yes No	5 58	0 28	0.125
**Pallor** Yes No	53 10	8 20	**0.000**
**Melena** **Hematemesis** **Rectal bleeding** **Hematemesis and Melena**	33 7 6 17	10 14 0 4	0.223
**Biological Variables**			
**Hemoglobin ≤ 9.5** Yes No	55 8	3 25	**0.000**
**Urea ≥ 9.7** Yes No	41 21	6 22	**0.000**
**Gastric Wash** Clear Attempted Hematic	36 8 9	12 4 3	0.33
**Endoscopic Variables**			
**Ulcer** Yes No	21 42	9 19	0.91

GBS was significantly higher in Group one (Table 4).

**Table 4:** The average score according to the need for a hemostasis procedure.

	Group 1	Group 2	p
N	63	28	
GBS	11.14	4.21	P: 0.001

For a cut-off of 9 points, sensitivity was 85.71% and specificity 92.86%. The positive predictive value was 96%. The negative predictive value was 74% (Table 5).

**Table 5:** Results for a threshold value of 9.

	Value	Interval
Sensitivity	85%	(74%–92%)
Specificity	92%	(75%–98%)
PPV	96%	(86%–99%)
NPV	74%	(56%–86%)

## 4 Discussion

To simplify decision-making, several clinical scores have been developed such as the Rockall score (RS), the Baylor score, the AIMS65 score, the Almela score, and the GBS [6,[10], [11], [12], [13]]. The most used ones are RS and GBS. These scores can be used to distinguish low-risk from high-risk patients, thus avoiding systematic admission to the intensive care unit or even routine hospitalization.

RS revealed positive predictive value for mortality and re-bleeding [14,15]. Therefore it could not predict endoscopic intervention [15]. The GBS is based only on clinical data, making it easy to calculate. Nevertheless, its clinical effectiveness remains unclear and there is little evidence available for North African population. Because of its simplicity, several teams (Table 6) have tried to validate it in their populations [5,[16], [17], [18], [19], [20]].

**Table 6:** Evaluation of the GBS by other teams.

Study	Cut off	Sensitivity	Specificity
Jarraya [7]	**< 7**	**96%**	**69%**
Koksal [8]	**>8**	**86%**	**69%**
Laursen [9]	**≤1**	**99%**	**39%**
Roberston [10]	**≤10**	**76%**	**83%**
Chandra [11]	**≤3**	**92%**	**33%**
Srirajaskanthan [12]	**≤2**	**100%**	**68%**

In this study, we analyzed the clinical outcomes in 91 patients. For a cut-off value of 9 points, the GBS was highly effective to predict the need for transfusion or hemostasis gesture with a positive predictive value of 96%.

Romagnolo J et al. demonstrated significantly fewer critical endoscopic lesions in patients of the low risk group according to a simplified GBS score (containing neither urea nor the presence of syncope). They also demonstrated that a low-risk GBS is associated with lower mortality and bleeding recurrence [21].

Stanley et al. [22] found in a prospective study that low-risk patients (GBS = 0) could be managed on an outpatient basis without developing any complications with a negative predictive value of 100%. They also confirmed that the GBS is better than pre and post-endoscopic Rockall scores in predicting mortality.

In a multicenter study of 1086 patients [23], the GBS identified low-risk patients (GBS = 0) with a 100% sensitivity but a specificity of only 6.3%. None of these patients required therapeutic intervention. Therefore, the authors recommended treating these patients on an outpatient basis with proton pump inhibitors (PPIs), as this strategy may reduce the cost of hospitalization.

Laursen et al. [18] found, in a study of 2305 patients, that the Glasgow Blatchford score, with a cut-off ≤ 1, had a high sensitivity (99.2%) in the prediction of low-risk patients. Indeed, only 4 cases among the 562 patients with a score less than or equal to 1, needed endoscopic treatment.

Similarly, in a study published by Mazoka et al. [24], the Blatchford score was significantly higher in the high-risk group. Based on a cut-off value of 2, the sensitivity and specificity of the Blatchford score were respectively 100% and 13%.

Jarraya et al. [16], showed that the GBS could identify low-risk patients with a sensitivity of 96% and a specificity of 69% for a cut-off <7.

In our study, only 5 patients had a score of 0. A GBS = 9 was defined as a discriminative value between high-risk patients (who had transfusions and hemostasis procedures) and low-risk patients (who received only injectable PPIs). Therefore, we identified high-risk patients with a sensitivity of 85.7% and a positive predictive value of 96.4%. This could simplify the management of digestive hemorrhages in emergency and surgical departments. Indeed, 75% of patients who had a score lower than 9 didn't have any therapeutic intervention, and 94% of those with a score greater than or equal to 9 required transfusions and/or hemostatic emergency procedures.

So, it appears that:

• A score inferior to nine should indicate injectable PPIs and hospitalization for close monitoring.
• For a score greater or equal to nine, it would be advisable to hospitalize patients in an intensive care unit for interventional endoscopy, surgery, and/or transfusions.

In summary, the main interest of the GBS lies in dispatching the patients between intensive care units for therapeutic intervention (if GBS> = 9) and ordinary hospitalization for surveillance (if GBS <9). It then makes it possible to rationalize the management of patients with digestive hemorrhage to identify those requiring hospital treatments (transfusion, endoscopic treatment, or surgery).

This study has some limitations that should be pointed out: This is a retrospective study and thus, the evidence level is limited. We consider that the endoscopic treatment rate is low and can be Moreover, the clinical outcomes recorded were limited to the events that happened during the hospital stay, which could also cause bias. There is potential for missed mortality and re-bleeding if patients presented to another health structure.

Our results showed that the GBS can be used to rationalize the management of gastrointestinal bleeding and is reliable in risk stratification.

Future prospective works with a larger sample would be more accurate to confirm our results.

## Ethical approval

Not required.

## Sources of funding

This research did not receive any specific grant from funding agencies in the public, commercial, or not-for-profit sectors.

## Author contribution

Houcine Maghrebi, Hazem Beji, Anis Haddad, and Amine Sebai did the conception and design of the work, the data collection, the data analysis and interpretation, and the writing of the manuscript. Samia Safraoui, Maroua Hafi, and Asma Laabidi participated in the writing of the manuscript. Mohamed Jouini and Montassar Kacem did the critical revision of the article and the final approval of the version to be published.

## Trial registry number

1.Name of the registry:

2.Unique Identifying number or registration ID:

3.Hyperlink to your specific registration (must be publicly accessible and will be checked):

## Guarantor

Houcine Maghrebi.

Hazem Beji.

## Consent

Not required.

## Provenance and peer review

Not commissioned, externally peer reviewed.

## Availability of data and materials

All relevant data and materials are provided with in manuscript.

## Declaration of competing interest

No conflicts of interest.
